# Harnessing Human Papillomavirus’ Natural Tropism to Target Tumors

**DOI:** 10.3390/v14081656

**Published:** 2022-07-28

**Authors:** Rhonda C. Kines, John T. Schiller

**Affiliations:** 1 Aura Biosciences, Cambridge, MA 02140, USA; 2 Laboratory of Cellular Oncology, Center for Cancer Research, National Cancer Institute, Bethesda, MD 20892, USA; schillej@mail.nih.gov

**Keywords:** human papillomavirus, heparan-sulfate proteoglycans, tumor therapy, photodynamic therapy, tumor immunotherapy

## Abstract

Human papillomaviruses (HPV) are small non-enveloped DNA tumor viruses established as the primary etiological agent for the development of cervical cancer. Decades of research have elucidated HPV’s primary attachment factor to be heparan sulfate proteoglycans (HSPG). Importantly, wounding and exposure of the epithelial basement membrane was found to be pivotal for efficient attachment and infection of HPV in vivo. Sulfation patterns on HSPG’s become modified at the site of wounds as they serve an important role promoting tissue healing, cell proliferation and neovascularization and it is these modifications recognized by HPV. Analogous HSPG modification patterns can be found on tumor cells as they too require the aforementioned processes to grow and metastasize. Although targeting tumor associated HSPG is not a novel concept, the use of HPV to target and treat tumors has only been realized in recent years. The work herein describes how decades of basic HPV research has culminated in the rational design of an HPV-based virus-like infrared light activated dye conjugate for the treatment of choroidal melanoma.

## 1. Introduction

There is a long and varied literature on the relationship of virus infection and cancer. Prominent examples include the determination of specific viruses as etiologic agents in specific cancers, the development of prophylactic vaccines to prevent these cancers, the initial identification and characterization of cellular oncoproteins and tumor suppressor proteins from studies of viral gene products, and the development of oncolytic virus-based cancer therapies. In this article we review a rather unique example of a virus/cancer relationship in which the characterization of the unusual early events in the HPV infection process involving tissue-associated and cellular proteoglycans have led to the finding of an unexpected tumor-specific tropism for the virus, and the exploitation of this knowledge to develop a potent and potentially broadly applicable treatment for cancers.

## 2. HPV Background

Papillomaviruses (PV) are small double-stranded DNA (dsDNA) viruses with a family with nearly 200 distinct types identified to date [1]. Papillomaviruses are not unique to humans having been found in over 50 animal species. The viral structure is composed of two proteins, L1 and L2, and it encapsidates a small ~8 kb genome encoding six “early” genes and the two “late” capsid genes. HPVs targets both cutaneous and mucosal basal epithelium and are regarded as the most prevalent sexually transmitted virus, although most infections are asymptomatic. HPVs are the primary etiological agent for several cancers, including almost all cervical cancers, and a substantial subset of anal, penile, vulvar, vaginal, and oropharyngeal tumors [2]. HPVs are grouped into “low risk” (LR) and “high risk” (HR) types loosely based on their presence in benign versus malignant disease. LR-HPV types such as types 1, 2, 6 and 11 can often be found in warts (common, genital, flat, verrucas, myrmecia), recurrent respiratory papillomatosis and lesions in individuals with epidermodysplasia verrucformis, though HR types have also been associated with these diseases in some cases [3,4,5,6]. A small group of 14 HPV types (16, 18, 31, 33, 35, 45, 51, 52, 56, 58, 59, 66 and 68) are classified as “high risk” based on their detection in HPV-associated cancers [2]. The viral oncoproteins E6 and E7 are the primary drivers of HPVs oncogenicity as they bind and effectively disable the tumor suppressor genes p53 and Rb, respectively [7,8,9]. Further, in addition to cancer, some studies have reported that HR-HPV infection can be detected in polyps (nasal and antrochoanal) and may impact both male and female fertility, though further exploration of these topics is warranted [10,11,12,13,14,15,16,17,18].

The study of HPV’s life cycle has met with roadblocks such as difficulty in producing native virions in cultured cells and establishing model systems to interrogate the HPV life cycle. However, advances in recombinant protein expression systems have greatly improved the tools available to PV researchers. When expressed as recombinant proteins, the L1 and L2 capsid proteins self-assemble into empty virus-like particles (VLPs). Importantly, L1 can self-assemble in the absence of L2, and this discovery was the basis for the current L1-based HPV prophylactic vaccines, Cervarix (16, 18), Gardasil (6, 11, 16, 18), Gardasil 9 (6, 11, 16, 18, 31, 33, 45, 52, 58), and Cecolin (16, 18) [19,20,21,22]. The advancement of VLP technology also afforded researchers the ability to study the early events of HPV binding and entry, however, it was the development of pseudovirus (PsV) technology that greatly facilitated the study of HPV intracellular trafficking followed by nuclear gene delivery. PsV are composed of both the L1 and L2 structural proteins encapsidating a plasmid mimicking the size of the dsDNA viral genome referred to as a “pseudogenome”. In the most frequently employed method, they are generated in monolayer cultured cells by co-transfection of plasmid DNA expressing the L1 and L2 capsid proteins that is too large to self-package, along with a smaller 6–8 kb plasmid, often encoding a reporter gene such as red fluorescent protein or luciferase, using a mammalian 293TT cell production system [23]. As the capsid proteins are made, they self-assemble and preferentially package the reporter plasmid as it mimics the size of the viral genome [24]. The final product allows for not only the study of the early binding and entry events of the virus, but also intracellular trafficking followed by nuclear delivery of the encapsidated plasmid and gene expression as a surrogate for the initial stage of infection.

## 3. HPV Infection Mechanism

A lingering problem that had vexed researchers studying HPV infection in the laboratory was the observation that HPV was unable to infect the known target cells, primary keratinocytes, *in vitro*, and virus binding and infection of intact epithelium in vivo proved elusive [25,26,27]. However, cultured cell lines, as well as their extracellular matrix (ECM), were capable of being bound by the virion. Over time, it was determined that the virus preferentially recognized and attached to specific modifications on heparan sulfate proteoglycans (HSPG) expressed on the cell surface and within the ECM that are not present on primary cells in culture [28,29,30]. These observations, coupled with the in vivo discovery that wounding of the upper epithelial layer led to virus binding and infection [26,31], resulted in the development of the model of the early events of HPV infection.

**Figure 1:**
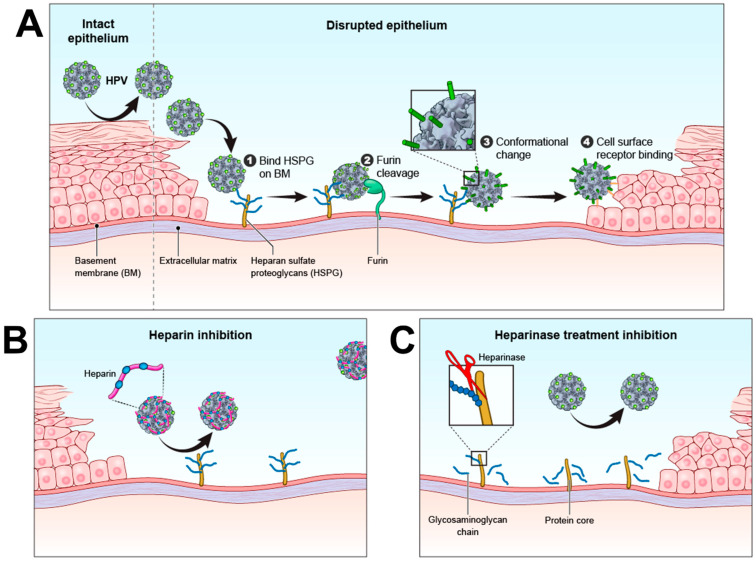
Mechanism of HPV attachment and infection. (**A**) HPV attaches to HSPG on the exposedbasement membrane (1). The L2 protein is then cleaved by furin (2) and the virion undergoes a conformation change (3) before attaching to a cell surface receptor (4). (**B**) depicts heparin inhibition of VLP attachment to basement membrane HSPG and (**C**) illustrates heparinase cleavage of glycosaminoglycan chains prevents HPV attachment. Human papillomavirus (HPV); heparan sulfate proteoglycan (HSPG); basement membrane (BM).

HPV cannot bind the apical surface of epithelial cells of intact tissues, instead it first requires access and attachment to the acellular basement membrane (BM) situated below the basal epithelial layer, a unique initial step in its infection process. Wounding or physical disruption of the epithelium must occur to expose the BM, and only then can the virus attach and initiate the early events of infection (Figure 1). The exposed BM is home to several molecules engaged in a signaling and structural interplay within the ECM, though most important for HPV, are HSPG and chondroitin sulfate proteoglycans (CSPG) [32]. The sulfation patterns of the polysaccharide chains of these proteoglycans undergo modifications promoting the various processes important during wound healing such as recruitment of growth factors, cell proliferation, and angiogenesis [33]. It is these specific modifications which provide the “molecular address” recognized by HPV in the context of a wound. Upon virus attachment to the BM-resident HSPG, the N-terminus of the L2 capsid protein undergoes a proteolytic cleavage event by furin or a related proprotein convertase [25,34,35]. This cleavage in turn alters the physical conformation of the viral particle such that previously occluded regions of L1 become exposed, and the virus is only then able to bind the cell surface of newly populating and dividing epithelial cells as they encroach on the wounded area. The virus can then enter the cell by an ill-defined L1-specific receptor and traffic to the nucleus leading to a productive infection, or in the case of the PsV, release of the “pseudogenome” as a surrogate for the first stage of infection indicating successful entry and DNA delivery to the nucleus (“pseudoinfection”) [35].

The use of HSPGs as part of its life cycle is not unique to HPV. Several other viruses, bacteria, parasites, yeast and even prions have been reported to rely on HSPG as minor or major factors in their binding and entry process (Table 1; reviewed in [36,37,38]. However, unlike HPVs, these microbes primarily bind directly to cell surfaces in vivo. As such, HSPGs are considered plausible targets in the prevention of infection and disease (e.g., heparin, heparin and HSPG mimetics and anti-syndecan 3; reviewed in Cagno et al. [37].

## 4. Proteoglycans

**Figure 2:**
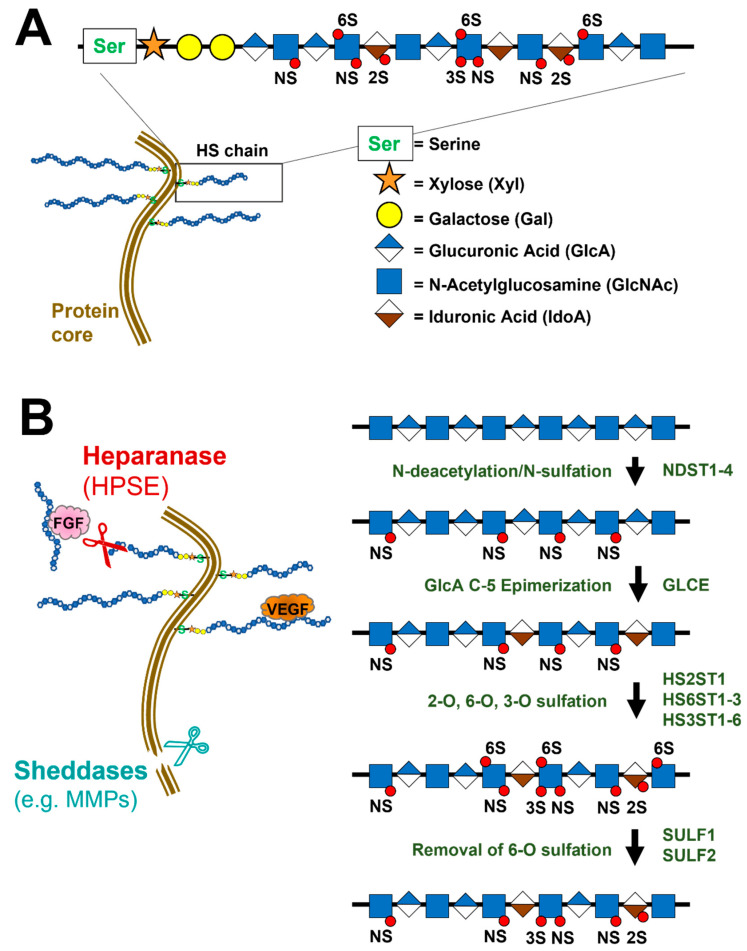
The structure and modifications of heparan sulfate proteoglycans (HSPG). (**A**) The general structure of an HSPG made up of a protein core and branched polysaccharide chains. (**B**) Examples of HSPG modifications such as sulfation patterns as well as remodeling by cleavage of the polysaccharide chains by heparinase or cleavage of the protein core by sheddases. N-sulfation (NS); heparan sulfate (HS); N-deacetylase/N-sulfotransferase (NDST); glucuronic acid epimerase (GLCE); O-sulfotransferases (HS2ST1, HS3ST, HS6ST); endosulfotransferases (SULF1, SULF2); heparanase (HPSE); matrix metalloproteinase (MMP).

Proteoglycans (PG) are complex molecules found ubiquitously across species and at their most basic are composed of a protein core with one or more covalently linked glycosaminoglycan (GAG) chains. They are important meditators of cell growth and tissue maintenance as they provide a scaffold to facilitate the local modulation and retention of growth factors, chemokines, cytokines and adhesion molecules, all key players in wound healing. Common GAGs are heparan sulfate (HS), chondroitin sulfate (CS), keratin sulfate (KS), dermatan sulfate (DS) and hyaluronic acid (HA). They have long polysaccharide chains composed of repeating disaccharide units of hexuronic acid (e.g., glucuronic acid (GlcA), iduronic acid (IdoA)) and hexosamine (e.g., N-acetyl glucosamine (GlcNAc)) (Figure 2A) [92,93,94]. The length, along with the sulfation and acetylation patterns on these chains, can vary thereby dictating the binding activity of the GAG and providing a functional “signature” of sorts. Remodeling of GAGs is dictated by environmental factors and often relies on localized enzymatic activity, primarily by sheddases such as matrix metalloproteinases (MMP), heparanase and sulfotransferases (Figure 2B) [95,96]. The modifications stemming from the remodeling process can, for example, enrich for growth factors such as basic fibroblast growth factor (FGF2) whose role in wound healing serves to activate fibroblasts and vascular endothelial cells enabling wound closure [33].

Through the years, a detailed view of the specific interaction of HPV with HSPGs has been elucidated, both *in vitro* and in vivo. HSPGs can exist as cell surface associated molecules (glypicans and syndecans) or secreted (perlecans) and, though some may exist on the cell surface, they can also be released following cleavage by heparanases and MMP to regulate the microenvironment. Importantly, it is within the wound healing milieu of the exposed BM that the modified HSPGs are present and provide the necessary recognition patterns for HPV attachment. Interestingly, even the basolateral surfaces of keratinocytes in wounded tissue are devoid of HPV-binding HSPGs stressing the importance of basement membrane exposure as the initial requirement for HPV attachment [26]. 

Using biochemical approaches, it was found that the HSPG modification patterns that HPV preferentially targets involve N-sulfated glucosamine, followed by 6-O GalNAc and 2-O GlcA/IdoA sulfation [30,32]. CSPG have also been implicated as an additional attachment factor for HPV under certain binding conditions and this recognition also relies upon 6-O sulfation of GalNAc [97]. Collectively these findings demonstrate the importance of the specific modification patterns that HPV relies upon to initiate its infectious process. HPV is not the only pathogen to rely on particular sulfation patterns for attachment and infection, for example herpes simplex virus type 1 (HSV-1) gD protein preferentially binds 3-O HS [58]; the capsid protein, pORF2 of hepatitis E virus (HEV) relies on 6-O sulfated syndecans for attachment to liver cells [46], and human cytomegalovirus (HCMV) associates with long, heavily sulfated HS chains exhibiting a preference for 6-O and 2-O sulfation [53]. 

## 5. HSPGs and Cancer

The importance of HSPGs in cancer progression cannot be understated as they play a profound role in all aspects of cancer biology from tumorigenesis to angiogenesis and metastasis. Much like the wound healing roles of modified HSPGs, tumors too require similar properties for growth factor recruitment and vascularization [98,99,100]. The sulfation patterns and chain lengths of proteoglycans can be modified within the tumor milieu by N-deacetylase/N-sulfotransferases (NDSTs), O-sulfotransferases (HS2ST1, HS3STs, HS6STs), endosulfotransferases (SULF1 and SULF2), heparanases (HPSE), sheddases and sulfatases (Figure 2B) [99]. Expression of these enzymes becomes aberrant in the tumor microenvironment because they are key to promoting and sustaining a malignant state. N-sulfation and 2-O sulfation are important sulfation sites for FGF2 attachment and promotion of endothelial proliferation and organization. 6-O sulfation, tightly regulated by the sulfotransferases, SULF1 and SULF2, is key to the stabilization of the ternary ligand-receptor complexes for both FGF2-FGFR1 and vascular endothelial growth factor (VEGF) and VEGFR, vital regulators of proliferation and angiogenesis [101,102,103,104,105,106]. 3-O GlcN sulfation is a rarely observed modification controlled by heparan sulfate 3-O sulfotransferase which has several isoforms. 3-O sulfation, and its associated sulfotransferase have been associated with increased tumorgenicity in several cancer models such as lung, pancreatic and leukemia [107,108,109].

Heparanases play a significant role in the controlling the length of HSPG chains such as those found on syndecan-1, freeing bound growth factors such as FGF2 and VEGF, thereby converting autocrine growth factor signaling within the environment to a paracrine one, promoting blood vessel formation and invasion [110,111]. Heparanase-mediated remodeling has also been associated with the epithelial to mesenchymal transition, a key feature in tumorigenesis and establishment of a pro-metastatic state attributed to increases in FGF2 and TGF-β signaling [99,112,113]. Reduction in heparanase expression as well as prevention of HSPG expression on tumor cells has been shown to reduce tumorgenicity in vivo, highlighting their importance in retaining a malignant phenotype as well as making them attractive targets for therapeutics [114,115,116]. 

## 6. Proteoglycans as Targets for Tumor Therapy

As tumors are heavily dependent upon GAGs and their respective modifying enzymes, these conserved molecules can serve as both therapeutic targets as well as biomarkers for disease staging and progression. Highly active heparinases can lead to shedding and release of GAGs along with their attached cargo, such as growth factors, resulting in more aggressive tumor growth. Shed syndecans have been demonstrated to be plausible biomarkers for predicting cancer progression in some studies, namely elevated serum levels of syndecan-1 are associated with a poor prognosis in bladder cancer, cervical cancer, and colon cancer, among others [117,118,119,120,121]. Kalscheuer et al. [122] demonstrated an association with upregulated levels of perlecan and poor prognosis in triple-negative breast cancer. 

The near ubiquitous use of varyingly modified proteoglycans by tumors affords researchers a wealth of cancer specific therapeutic targets. The observation that soluble heparin, which commonly serves as an anti-thrombotic drug, reduced tumor burden and metastasis was the start of the exploitation of proteoglycans for tumor therapy [123]. Now, heparin and heparan sulfate mimetics such as Muparfostat (heparinase competitor), Necuparinib (reduces MMP activity), and Roneparstat (heparinase competitor) are in various stages of clinical development in an attempt to impact tumor growth and spread (Table 2). GAG targeted peptides (e.g., Arginylglycylaspartic acid (*RGD* motif)), nanoparticles, antibodies and modified pathogens carrying toxic payloads and more recently CAR-T cells, are also in various stages of clinical development (Table 2) [124,125,126].

The tumor ECM can serve as a sink or barrier inhibiting translocation of oncolytic viruses or other viruses targeting tumors thereby preventing broader tumor distribution. To overcome this, tumor targeting viruses can be engineered to express genes for ECM degrading enzymes such as hyaluronidase, chondroitinase and MMPs which can facilitate the localized breakdown of tumor ECM resulting in enhanced spread of the oncolytic virus [127,128]. For these aforementioned reasons, targeting the GAG modifying enzymes or the tumor associated proteoglycans is a burgeoning field of tumor therapy.

## 7. HPV Capsids as a Tumor Therapeutic

The similarity between the HSPG modifications found on tumors and those on exposed basement membranes makes HPV and attractive tool for tumor-directed therapy. HPV capsids were found to bind a wide variety of human tumor types in a screen of the NCI-60 panel of human tumor cell lines, particularly epithelial derived cancers such as ovarian, lung and breast [171]. Using biochemical assays to decipher HPVs tumor binding characteristics *in vitro*, it was noted that heparinase treatment of tumor cells abrogated HPVs binding ability and the same basement membrane-associated HSPG N-, 6-O and 2-O sulfation patterns were responsible for HPV tumor targeting. This HSPG targeting was verified in vivo using *i*-carrageenan, a heavily sulfated polysaccharide, to inhibit HPVs binding and infection of human tumor cells. Importantly, the HPV capsids did not detectably bind the apical surfaces of a wide variety of intact tissues [171]. In considering the use of HPV as a cancer therapeutic, it is important to note that the VLP can be easily modified to carry a “payload” such as a dye without altering its tumor targeting ability. In addition to modifications to the VLP itself, using pseudovirus technology, the virus can specifically deliver nucleic acids to tumor cells resulting in locally expressed genes within the tumor microenvironment minimizing off-target or systemic effects [171,172,173].

In addition to their tumor specific HSPG targeting capability, the 55 nm HPV VLP fits into a particular niche of nano sized particles that preferentially collect in tumors due to leaky vasculature. As tumors grow, their interior becomes hypoxic forcing the tumor to undergo neovascularization, but in most cases, these newly generated blood vessels are disorganized and porous, allowing for preferential extravasation of particles of 40–120 nm to passively accumulate within the tumor, an observation referred to as the EPR effect (enhanced permeabilization and retention) [174,175]. With its size and tropism for tumors, the HPV VLP is a natural vehicle for cancer therapeutics that may be exploited in many ways.

### 7.1. Gene Delivery

While the HPV PsV has proven a useful tool for studying the early stages of HPV binding, it also affords researchers a useful vector for gene delivery to tumors. In initial studies involving intravenous delivery of HPV PsV packaging reporter plasmids, spontaneous and orthotopically implanted tumors in syngeneic and xenograft murine models of bladder, ovarian, and lung cancer were specifically transduced [171,172,173]. Importantly, there were no off-target gene transduction events detected, indicating that the tumor targeting was specific. In the human NCI-H460 non-small cell lung cancer orthotopic tumor model, PsV infection (as noted by transduction of the firefly luciferase gene) was observed in a subset of animals in regions other than the lungs. Upon microscopic assessment, it was determined that intravenous delivery of HPV PsV had targeted metastatic tumors in lymph nodes, kidneys, and ovaries [171]. Using HPV PsV to target orthotopic ovarian and bladder tumors, Hung et al. [172] and Hojeij et al. [173] demonstrated delivery of a gene for thymidine kinase (TK), a commonly used “suicide” gene that activates the prodrug ganciclovir. Both groups reported an impact on tumor growth and tumor burden specific to the animals receiving the TK gene delivered by HPV PsV followed by ganciclovir treatment. However, a potential limitation of this approach is that HPVs complete their infectious process only in actively dividing cells, and tumors generally have a subpopulation of quiescent cells. This constraint might be less critical if the PsV were used in an adjuvant capacity, for example to deliver immune modulatory genes to a tumor or to express cytokines and chemokines to generate a chemoattracting gradient to enhance recruitment of immune cells to the tumor milieu.

### 7.2. HPV Virus-like Particles as Drug Conjugates

**Figure 3:**
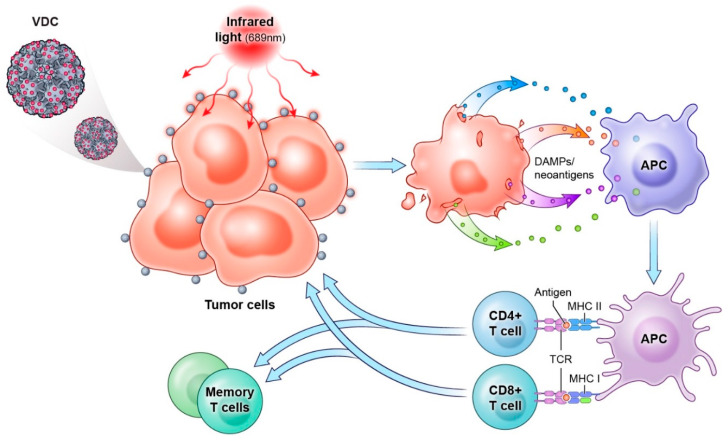
Mechanism of AU-011 (belzupacap sarotalocan) mediated tumor killing. AU-011 binds to the surface of tumor cells and is activated by near infrared light. The killed tumor cells release DAMPs and neoantigens into the local tumor milieu resulting in uptake by and activation of antigen presenting cells. Both CD4+ and CD8+ T cells are activated and are necessary for localized tumor control and long-term protection from tumor re-challenge. Virus-like drug conjugate (VDC); damage associated molecular patterns (DAMPs); antigen presenting cells (APC); major histocompatibility complex (MHC); T cell receptor (TCR).

A second general strategy to overcome the infection limitation of HPVs is to deliver drugs that only require capsid binding, which occurs similarly in dividing and nondividing tumor cells. The most advance example of this approach is AU-011 (belzupacap sarotalocan), a virus-like drug conjugate (VDC) composed of a modified HPV16 VLP (L1 and L2 capsid proteins) conjugated with the phthalocyanine photosensitizing dye, IRDye700DX [176,177]. The L1 amino acid sequence has been modified to reduce interaction with pre-existing neutralizing antibodies, but this modification has no impact on the tumor associated HSPG targeting. When bound to tumor cells and photoactivated by 689–690 nm near-infrared (NIR) light, AU-011 releases reactive oxygen species and causes physical disruption of the tumor cell membrane resulting in rapid and acute, necrotic cell death (Figure 3). Using *in vitro* mixed cell assays in which human tumor cells were combined with HSPG deficient cells (pGSA-745) and exposed to AU-011 followed by NIR light treatment, extensive cytotoxicity for the tumor cells was observed, while simultaneously sparing the HSPG negative cells. AU-011 mediated tumor cytotoxicity also leads to the release of tumor neoantigens, and damage associated molecular patterns (DAMPs), such as ATP, cell-free DNA, HMGB-1, and the subcellular re-localization of ER proteins HSP70 and calreticulin to the surface of the cell, resulting in a potently immunogenic tumor milieu capable of stimulating cell-mediated anti-tumor immunity (Figure 3) [178].

In vivo murine syngeneic tumor models, TC-1 and MB49-luciferase have been used to better understand AU-011’s efficacy and its potential to generate anti-tumor immunity. A single intravenous administration followed by NIR exposure of the tumor resulted in >50% complete response rates with animals remaining tumor free up to 100 days post-treatment. Tumor-free animals were re-challenged with corresponding tumor cells and again >50% were protected from tumor outgrowth indicating that a long-term anti-tumor immune response had been induced. When AU-011 was combined with checkpoint inhibitors, anti-CTLA-4 or anti-PD-1, tumor-free survival rates were increased to 100% and 75%, respectively, and >70% of tumor-free animals were protected from tumor re-challenge after 100 days. Induction of both CD4+ and CD8+ anti-tumor T cells was a key contributing factor to AU-011’s efficacy, both at the time of treatment, and for maintaining long-term protection [178]. 

Inherent in their nature, VLPs will bind and be taken up by antigen presenting cells of the hosts immune system. In human peripheral blood lymphocytes, the VLPs avidly bind neutrophils, monocytes, macrophages, dendritic cells and B cells, but not T or NK cells [179]. Within a tumor resides suppressive populations of these cells, such as tumor associated macrophage (TAM) and myeloid-derived suppressor cells (MDSC), primarily serving to shield the tumor from host immune responses. AU-011 can bind and kill these cells within the tumor microenvironment, lending further credence to its ability to generate a potent immunogenic tumor microenvironment [178].

AU-011 is currently being assessed for it use as a first-line treatment for choroidal melanoma, a disease with minimal early interventions [180]. Pre-clinical data demonstrated that intravitreal or suprachoroidal injection into the eyes of rabbits bearing choroidal melanoma tumors resulted in AU-011 tumor distribution and dose dependent efficacy upon NIR treatment (as measured by tumor necrosis and tumor control) [177,181,182]. A Phase 1/2b dose escalation study (Clinical Trial #NCT03052127) examining intravitreal administration of AU-011 to patients with small primary choroidal melanomas resulted in minimal adverse events, with inflammation and increases in intraocular pressure being the most common, though not unexpected due to AU-011′s potent immune stimulatory capabilities and mechanism of action. Importantly, there were no dose limiting toxicities observed and, for those patients experiencing inflammation or intraocular pressure, symptoms could be managed with steroids and ocular hypertensives. Interim data suggests ≥55% tumor control across all dosing groups including a subtherapeutic dose and regimen and ≥87% vision preservation at the time of interim analysis (12-month median follow-up) [182]. Vision preservation is critical as current standard of care for choroidal melanoma is typically radiotherapy such as plaque brachytherapy which often results in vision loss as a result of complications such as retinopathy, cataracts and neovascular glaucoma [180]. An additional Phase 2 safety and efficacy trial is underway (Clinical Trial #NCT04417530) to deliver AU-011 directly into the suprachoroidal space of the eye with the idea of directly localizing the drug within the tumor compartment rather than relying on it to distribute throughout the vitreous and cross the retina and retinal pigment epithelium (RPE) to bind to the tumor after intravitreal injection. Future studies are planned to examine the use of AU-011 to treat other cancers that metastasize to the choroid as well as to examine its applicability for treatment of non-muscle invasive bladder cancer.

## 8. Conclusions

The use of viruses and nanocarriers is an expanding field in cancer therapy seeking to deliver sensitizing drugs (phototherapy, thermotherapy, ultrasound), chemotherapeutics, immune stimulators, or cytotoxic genes. While advances in targeted tumor therapies have revolutionized cancer research in the past 20 years, many rely on personalized therapy (T cells) or tumor-type restricted surface ligands (CAR-T, mAbs). Tumor associated modified proteoglycans provide a target that can be found on a breadth of cancer types. Unlike HPV capsids that possess natural targeting capabilities, proteoglycan targeted nanoparticle therapies require modifications with ligands, targeting peptides or scFv that recognizes the particularly modified tumor proteoglycans to deliver their payload. The HPV capsid binding studies revealed an unanticipated commonality of HSPG-specific modifications across many cancer types. Therefore, a clear advantage HPV has over many other supramolecular candidates is that it does not require any modification to preferentially recognize tumors, making it a broadly applicable biologic for cancer therapy, both in terms of the type of payload it can deliver and the types of cancers it can target.

## 9. Patents

RCK-US Patent 9,855,347 B2 (issued) for virion-derived nanospheres for selective delivery of therapeutic and diagnostic agents to cancer cells; U.S. Patent 10,117,947 B2 (pending) for virus-like particle conjugates for diagnosis and treatment of tumors; WO/2018/191363 (pending) for targeted combination therapy. JTS-US Patent 10,117,947 B2 (issued, licensed, and with royalties paid from Aura Biosciences); US Patent 10,188,751 B2 (issued, licensed, and with royalties paid from Aura Biosciences); US Patent 8,990,290 B2 (issued, licensed, and with royalties paid from Aura Biosciences).

## Figures and Tables

**Table 1:** Pathogens utilizing proteoglycans for host infection.

Pathogen	Ligand	Reference
*Viruses*		
Adenovirus (AdV)	fiber	[39,40]
Dengue virus	envelope	[41]
Hepatitis B virus (HBV)	L-envelope	[42,43]
Hepatitis C virus (HCV)	E2 envelope	[44,45]
Hepatitis E virus (HEV)	ORF2 capsid protein	[46]
Human immunodeficiency virus (HIV)	gp120, Tat	[47,48,49,50]
Human cytomegalovirus (HMCV)	gB	[51,52,53]
Human papillomavirus (HPV)	L1 capsid potein	[28,30,32]
Herpes simplex virus type 1 (HSV-1)	gB, gC, gD	[54,55,56,57,58,59]
Herpes simplex virus type 2 (HSV-2)	gB, gC	[54,59,60,61]
Merkel cell polyomavirus (MCPyV)	VP1	[62,63]
Severe acute respiratory syndrome coronavirus type 2 (SARS-CoV-2)	spike	[64,65]
Varicella zoster virus (VZV)	gB	[59,66]
		
*Parasites*		
*Giardia lamblia*	Alpha-1 giardin	[67]
*Plasmodium falciparum*	BAEBL, VAR2CSA, CS	[68,69,70]
*Toxoplasma gondii*	SAG3, ROP2, ROP4, GRA2, SAG1	[71,72,73,74]
*Trypanosoma cruzi*	Heparin binding proteins (HBP)	[75,76,77]
		
*Bacteria*		
*Bordatella pertussis*	FHA	[78]
*Helicobacter pylori*	VacA	[79,80]
*Listeria monocytogenes*	ActA	[81]
*Mycobacterium tuberculosis*	HA	[82]
*Neisseria gonorrhoaea*	Opa	[83,84]
		
*Other*		
Candida albicans	n.d.	[85,86,87]
*Malassezia* spp.	n.d.	[87]
Prion	PrP	[88,89,90,91]

**Table 2:** Proteoglycan targeted tumor therapies.

Name	Target/Mechanism	Reference
*Antibodies*		
HN3	PE38, PE24 conjugates targeting glypican-3	[129,130]
GC33	ADCC; targeting glypican-3	[131,132]
YP7	PE38, Duocarmycin, IRdye700DX conjugates; pyrrolobenzodiazepine dimer; targeting glypican-3	[129,133,134,135]
32A9	PE24 conjugates targeting glypican-3	[136]
ERY974	bi-specific antibody agains glypican-3 and CD3	[137]
D4 (camel)	PE38-conjugated camelid nanobody targeting glypican-1	[138]
LH7	PE38-conjugated human single domain anti-glypican-2	[139]
*CAR-T*		
GC33	Glypican-3	[140,141]
hYP7	Glypican-3	[142]
32A9	Glypican-3	[136]
Y035	Glypican-3	[143]
LH7	Glypican-2	[139]
*Small molecule/peptide mimics/false substrates*		
Guanidinylated neomycin (Gneo)	LMW HS binding peptide carrying saporin	[144]
Synstatin (SSTN)_92–119_	Peptide blocks syndecan-1/IGF1R complex-blocks integrin signaling and VEGFR2 activation	[145]
RGWRGEKIGN peptide	HS binding peptide blocks FGF2/HS binding	[146]
NT4	General heparin, HSPG, CSPG mimetic (tetra-branched polypeptide); interferes with cell migration; delivers paclitaxel	[147,148,149]
OKN-007	Sulfatase-2 inhibitor	[150]
PI-88 (muparfostat)	Heparanase inhibitor (heparin mimetic); interferes with VEGF, FGF1, FGF2 leading to reduction in angiogenesis and sulf1 and sulf2 activity	[151,152]
Suramin analogs	Heparanse inhibition; inhibits degradation of ECM and blocks angiogenic events by preventing release of FGF from ECM HS	[153]
PG545	HS mimetic; blocks heparanase activity; prevents growth factor release and activation	[154,155,156]
M402 (neuparanib)	HS mimetic; inhibits HS interactions and activity of VEGF, FGF2, SDF-1α, P-selectin, and heparanase	[157]
SST0001 (roneparstat)	Split heparin; inhibits heparanase, downregulates HGF, VEGF, and MMP-9 expression and suppresses angiogenesis	[158]
Xylosides	Blocks GAG biosynthesis	[159,160]
*Nanoparticles/Pathogens*		
Ad5	Fiber modified to bind HSPG (bypass CAR)	[161]
hyaluronic acid micelle nanocarrier	Hyaluronic acid nanocarrier targeting CD44; incorporate doxorubicin and cisplatin	[162]
rVAR2CSA	Targets oncofetal CS (CD44, CDPG4; syndecan-1); conjugated with diptheria toxin or hemiasterlin	[163,164]
liposomes	Composed of glypican-3 targeting peptide incorporating sorafenib; GAG binding peptide incorporating doxorubicin	[165,166]
metal conjugates	HSPG targeted peptide and glypican-3 antibody delivering Fe_3_O_4_ for imaging; Gold nanocluster with gadolinum conjugated to anti-glypican-1; Gold nanocages incorporating gemcetabine conjugated to anti-glypican-1 for theranostics	[167,168,169,170]
